# 4-phenylbutyrate exerts stage-specific effects on cardiac differentiation via HDAC inhibition

**DOI:** 10.1371/journal.pone.0250267

**Published:** 2021-04-21

**Authors:** Yanming Li, Xiaofei Weng, Pingping Wang, Zezhao He, Siya Cheng, Dongxing Wang, Xianhui Li, Guanchang Cheng, Tao Li

**Affiliations:** 1 Department of Cardiology, Huaihe Hospital of Henan University, Kaifeng, Henan Province, China; 2 School of Medicine, Hunan Normal University, Changsha, Hunan, China; 3 Department of Health Service, Logistics College of People’s Armed Police Force, Tianjin, China; University of Tennessee Health Science Center College of Medicine Memphis, UNITED STATES

## Abstract

4-phenylbutyrate (4-PBA), a terminal aromatic substituted fatty acid, is used widely to specifically attenuate endoplasmic reticulum (ER) stress and inhibit histone deacetylases (HDACs). In this study, we investigated the effect of 4-PBA on cardiac differentiation of mouse embryonic stem (ES) cells. Herein, we found that 4-PBA regulated cardiac differentiation in a stage-specific manner just like trichostatin A (TSA), a well-known HDAC inhibitor. 4-PBA and TSA favored the early-stage differentiation, but inhibited the late-stage cardiac differentiation via acetylation. Mechanistic studies suggested that HDACs exhibited a temporal expression profiling during cardiomyogenesis. *Hdac1* expression underwent a decrease at the early stage, while was upregulated at the late stage of cardiac induction. During the early stage of cardiac differentiation, acetylation favored the induction of *Isl1* and *Nkx2*.*5*, two transcription factors of cardiac progenitors. During the late stage, histone acetylation induced by 4-PBA or TSA interrupted the gene silence of *Oct4*, a key determinant of self-renewal and pluripotency. Thereby, 4-PBA and TSA at the late stage hindered the exit from pluripotency, and attenuated the expression of cardiac-specific contractile proteins. Overexpression of HDAC1 and p300 exerted different effects at the distinct stages of cardiac induction. Collectively, our study shows that timely manipulation of HDACs exhibits distinct effects on cardiac differentiation. And the context-dependent effects of HDAC inhibitors depend on cell differentiation states marked by the temporal expression of pluripotency-associated genes.

## Introduction

Cardiac differentiation of pluripotent stem cells *in vitro* mimics cardiomyogenesis *in vivo*, exhibiting several developmental stages. Well-established protocols to transform stem cells into cardiac cell lineages are usually based on the stepwise stages of cardiomyogenesis *in vitro*. In addition, cardiomyogenesis is a closely orchestrated process integrating multiple signaling pathways and transcription factors in a synergic manner. A variety of signaling pathways is involved in the stepwise process of cardiac specification. Wnt, Notch, BMP, TGFβ, and MAPK pathways exert temporal effects on cardiomyogenesis [1–4]. Definite evidences prove that Wnt and Notch signals generally promote the early-stage differentiation, while impose negative effects on the later stages of cardiac differentiation [1, 5]. Therefore, the optimization of cardiac induction need a profound understanding about the stepwise cardiac differentiation, and then an elaborate steering.

The temporal supplementation of distinct growth factors (such as BMP2, BMP4, Activin A, bFGF, FGF10, and Wnt3a), has also been found to elevate the induction efficiency of spontaneously beating cardiomyocytes [6–11]. However, low throughput and enormous expenditure restrict the large-scale application of protein inducing reagents. Small molecules, pharmacologically blocking or activating specific signaling pathways, are an alternative approach to achieve a satisfactory quantity and purity of pluripotent stem cells-derived cardiomyocytes. It well established that ascorbic acid (AA) can robustly and reproducibly evoke cardiac differentiation when pluripotent stem cells are allowed to aggregate in differentiation medium and form embryoid bodies (EBs) in which early embryonic cell lineages develop [12]. KY02111 and XAV939, small molecules inhibiting Wnt signaling, respectively promoted differentiation of induced pluripotent stem (iPS) cells and ES cells to cardiomyocytes [13–15]. Dorsomorphin, a selective small molecule of BMP inhibitor, robustly enhanced cardiomyogenesis when its administration was limited to the initial stages of ES cell differentiation [3]. The Notch inhibitor DAPT facilitated the maturity of cardiac mesoderm and the formation of cardiac progenitors [5]. SB203580, a specific p38 kinase inhibitor, augmented the differentiation of ESCs into cardiomyocytes by facilitating early mesoderm formation at low concentrations [16].

4-phenylbutyrate (4-PBA) is a terminal aromatic substituted fatty acid and is usually used as an inhibitor of endoplasmic-reticulum (ER) stress. Meanwhile, 4-PBA also functions as an inhibitor of histone deacetylase (HDAC). It is unknown whether 4-PBA affects cardiac differentiation via the modulation of ER stress. In this study, we evaluated the effects of 4-PBA on cardiac differentiation of mouse ES cells. Interestingly, induction of ER stress by tunicamycin (TM) promoted cardiac differentiation at the early and late stages. In contrast, 4-PBA favored the early-stage differentiation, but inhibited the late-stage cardiac differentiation. Further investigation revealed that 4-PBA displayed similar stage-specific effects on cardiac differentiation as trichostatin A (TSA), a histone deacetylase inhibitor. Indeed, HDAC1 exhibited a temporal expression profiling during cardiomoyogenesis. At the early stage of cardiac differentiation, 4-PBA and TSA promoted histone acetylation, thereby enhanced the expression of *Isl1* and *Nkx2*.*5*, two of the early markers for cardiac progenitors. At the late stage of cardiac differentiation, histone acetylation interrupted the depletion of OCT4, a key determinant of self-renewal and pluripotency. Therefore, the treatment with 4-PBA and TSA at the late stage attenuated the expression of cardiac-specific contractile proteins, decreasing the generation of spontaneously beating cardiomyocytes. Overall, our study shows that inhibition of HDACs via 4-PBA or TSA can modify epigenetic program to affect cardiac differentiation in a stage-dependent manner.

## Materials and methods

### Reagents and plasmids

Ascorbic acid (AA), 4-PBA, TM, TSA, and SP600125 were purchased from Sigma-Aldrich or Selleck Chemicals, respectively. Antibodies that were used in the present study included troponin T (A4914, Abclonal), JNK (#9252, Cell Signaling), phospho-JNK (Thr183/Tyr185) (#4668, Cell Signaling), HDAC1 (sc-81598, Santa cruz), ISL1 (ab-109517, Abcam), NKX2.5 (sc-376565, Santa cruz), OCT4 (Santa cruz), SOX2 (sc-365823, Santa cruz), NANOG (sc-134218, Santa cruz), histone H3 and acetylated-H3K9(ab1791 and ab10812, Abcam). pCMV-p300 was a gift from Dr T. Kouzarides (University of Cambridge, Cambridge).

### Cell culture

The mouse ES cells (E14tg2a) were purchased from ATCC (American Type Culture Collection, USA). ES cells were maintained in Dulbecco’s Modified Eagle’s Medium (DMEM, Gibco, USA) supplemented with 15% fetal bovine serum (FBS, Hyclone, USA), 1000 U/mL leukemia inhibitory factor (LIF, Sigma, USA), 100 nM β-mercaptoethanol (Sigma, USA), 1% non-essential amino acids (Gibco), 100 units/ml penicillin, and 100 mg/ml streptomycin (Beyotime, Jiangsu, China) without feeder cells. Cells were cultivated in 5% CO_2_ atmosphere at 37°C, and routinely passaged every 2 days using trypsin.

### Cardiac induction

The mES cells were induced into cardiac cell lines *in vitro* through the formation of embryonic body (EB) according to the previous report [17]. To generate EBs, mES cells were detached by trypsin treatment and transferred onto low-attachment plates (Jing Dian, Qingdao, China). Cells were resuspended and aggregated to form EBs in induction medium containing 100 nM AA without β-mercaptoethanol and LIF factors. The medium was refreshed every two or three days. After suspension induction for 5 days, EBs were attached to plates coated with 0.1% gelatin for adherent culture in the presence of AA for 4 days. And then cells were maintained in DMEM medium plus 15% FBS. The ratio of spontaneously contracting EBs to the total number of plated EBs was counted at indicated days, and nine random visual fields were selected for statistical analysis.

### Real-time PCR

Total RNA was extracted using Trizol reagent (Invitrogen). 2 μg of total RNA from each sample was used for reverse transcription, the PCR was carried out as previously described. For real-time RT-PCR, SYBR Green real-time Master Mix (Toyobo, Japan) and the ABI7300 Real Time PCR were used (Applied Biosystems, USA). Target gene expression levels were normalized by calculating the Targets/18S expression ratio (2^-ΔΔCt^). Primers for real-time PCR were listed in the S1 Table.

### Western blot

Total protein extracts were obtained with lysis buffer (150 mM NaCl, 10 mM Tris-HCl, 5 mM EDTA, 0.1% SDS, 1% sodium deoxycholate, 1% Triton X-100, pH 7.2) containing protease inhibitor cocktail (Roche, USA). Proteins were separated by electrophoresis on 8–15% SDS-polyacrylamide gels, transferred to PVDF membranes (Millipore, USA), and incubated with corresponding primary antibodies. The blots were next incubated with corresponding peroxidase-conjugated secondary antibodies and visualized with the enhanced chemiluminescence kit (Millipore). The membrane was also probed for glyceraldehyde-3-phosphate dehydrogenase (GAPDH) as a loading control. The blots shown were representative of three independent experiments.

### Immunofluorescence

For immunofluorescence, ES cells at indicated days were fixed in 3.7% (w/v) formaldehyde for 15 min, followed by permeabilization with 0.1% Triton-100. Subsequently, cells were blocked with 5% BSA in PBS for 1 h at room temperature and stained with the primary mouse monoclonal antibody (1:200) against troponin T, or NKX2.5 overnight at 4°C. The next day, cells were incubated with TRITC-conjugated goat anti-rabbit IgG (1:200). Nuclei were counterstained with Hoechst 33342 (Sigma) for 5 min at room temperature. Images were viewed under an Olympus FV1000 confocal laser scanning microscope.

### Plasmid transfection

The mES cells were transfected with HDAC1 (Sino Biological Inc, Beijing, China) or p300 plasmids for gene overexpression. Cells were seeded at a density of 10×10^4^ per well in a 24-well plate and transfected using Lipofectamine™ Stem Transfection Reagent (Invitrogen) according to the manufacturer’s protocols. pEGFP vector was used as a control. Differentiating cells in EBs were digested into single cells by Accutase (Invitrogen), and reseeded for transient transfection. After transfection, the cells were subjected for EB formation and/or cardiac induction, and harvested at the indicated days. All the transfections were performed at least three times.

### Methylation analysis

Methylation analysis of the *Oct4*, *Sox2*, *Nkx2*.*5*, *Myl7* promoters was carried out using bisulphite modification and mass spectrometric analysis in BioMiao Biological Technology (Beijing). Genomic DNA was bisulphate-converted and amplified by PCR by using primers (S2 Table). Primers for all the genes were designed to cover the region with the most CpG sites. The selected amplicon was located in the promoter region of the gene. The PCR fragments are quantitated by mass spectrometry. The mass spectra were collected using a MassARRAY Compact MALDI-TOF (SEQUENOM, BioMiao Biological Technology, Beijing) and the spectra’s methylation ratios were generated by the EpiTYPER software.

### Chromatin immunoprecipitation (ChIP) assays

ChIP assays were performed on these cells using the EZ ChIP kit (Millipore, Los Angeles, CA, USA) according to the manufacturer’s instructions. Briefly, alter cells were collected and the cross-linked chromatin was sheared to an average DNA fragment length of 100–800 bps, immunoprecipitation was performed using 5 μg of acetyl-histone H3K9 antibodies. Normal IgG was used as a negative control. The precipitated DNA was amplified by real-time PCR. The data are normalized on the basis of the corresponding input. Primers were used to amplify the promoters of target genes and in the S3 Table.

### Statistic analysis

All experiments were performed at least 3 times to assure reproducibility and data were displayed as mean ± standard deviation (SD). Comparisons between groups were analyzed by Student’s *t* test or ANOVA. The significance was analyzed with SPSS13.0 software, and a value of *P*<0.05 was considered statistically significant.

## Results

### 4-PBA and TM differently affect cardiac differentiation

**Fig 1:**
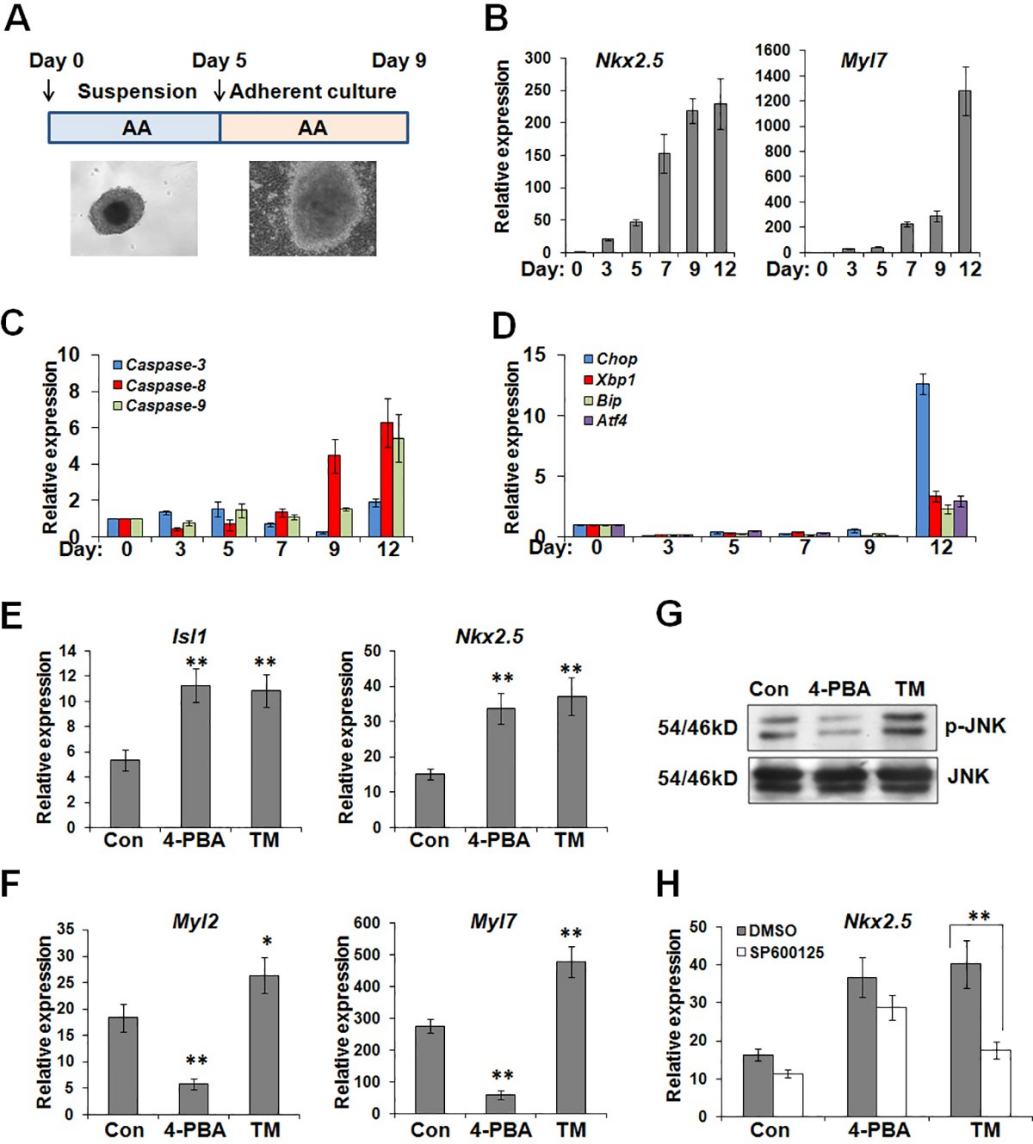
4-PBA and TM differently affect the early- and late-stage cardiac differentiation. (A) Schematic diagram of differentiation protocol. mES cells were induced into cardiomyocytes using 100 nM AA with a two-stage induction protocol. (B) Real-time PCR was conducted to evaluate the expression of *Nkx2*.*5*and *Myl7* during the stepwise process of cardiac induction. (C) and (D) The expression fluctuation of *Caspase-3*, *-6*, and *-9*, as well as ER stress-associated genes *Chop*, *Xbp1*, *Bip*, and *Atf4* during cardiomyogenesis was estimated by real-time PCR. (E) After the treatment of TM (0.5 μg/ml) and 4-PBA (1 mM) during the early stage of induction (day1-5), the expression of *Isl1* and *Nkx2*.*5* was estimated by real-time PCR. *P<0.05, **P<0.01 compared with the control. (F) After the treatment of TM and 4-PBA during the late stage of induction (day6-9), the expression of *Myl2* and *Myl7* was estimated by real-time PCR. *P<0.05, **P<0.01 compared with the control. (G) The cell lysates were extracted from differentiating ES cells treated with TM or 4-PBA during the early stage of induction (day1-5), and subjected for western blot with antibodies against the total and phosphorylated JNK kinases. (H) Differentiating ES cells were treated 5 μM JNK inhibitor SP600125 in the presence of TM or 4-PBA during the early stage of induction (day1-5), and subjected for real-time PCR. **P<0.01.

A two-stage protocol was used to induce cardiac differentiation and examine the expression profiling of several genes associated with cellular stress responses (Fig 1A). During suspension culture, ES cells underwent early-stage differentiation; cardiac progenitors began to occur and the cells expressed the early cardiac transcription factors such as *Nkx2*.*5*. During adherent culture, stem cells accomplished late-stage differentiation, and transformed into mature cardiomyocytes expressing cardiac contractile genes such as *Myl7* (Fig 1B). Subsequently, we assessed the expression profiling of genes associated with cellular responses under oxidative stress and ER stress. *Caspase*-3 expression was slightly upregulated at the early stage (day 3 and 5) of cardiac differentiation, and then underwent a decrease and a subsequent increase at day 12 (Fig 1C). By comparison, after stem cells specialized into beating cardiomyocytes, the expression of *Caspase*-*8* and -*9* was greatly enhanced at day 12. ER resident chaperones *Chop* and *Bip*, two markers of ER stress, were strongly repressed during cardiac induction, while a great increase in expression could be observed at day 12 (Fig 1D). *Atf4* and *Xbp1*, transcription factors of the genes for Chop and other ER stress-related genes, also displayed a remarkable enhancement in expression at day 12. Taken together, these results implicate that ER stress-associated genes exhibit distinct expression profiling during cardiomyogenesis.

Subsequently, we investigated the effects of TM and 4-PBA on cardiac differentiation. TM can induce ER stress through unfolded protein response. 4-PBA is related to the inhibition of ER stress. As shown in Fig 1E, both of TM and 4-PBA at the early stage of induction (day1-5) had a promotion on cardiomyogenesis, as evidenced by enhanced expression of *Isl1* and *Nkx2*.*5*, two of the early markers of cardiac progenitors. Interestingly, 4-PBA inhibited the late-stage cardiac differentiation (day6-9), leading to a great decreased expression of cardiac contractile genes, *Myl2* and *Myl7* (Fig 1F). In contrast, TM also promoted the late-stage cardiac differentiation. As oxidative stress facilitates cardiac differentiation via JNK cascade, we then estimated the effects of TM and 4-PBA on JNK activation. After the cocultivation with TM during differentiation day 1–5, JNK phosphorylation was effectively enhanced (Fig 1G). The supplement of specific JNK inhibitor SP600125, completely abolished TM-induced increase in *Nkx2*.*5* expression (Fig 1H). SP600125 could not interrupt 4-PBA effects on *Nkx2*.*5* expression. Collectively, these data showed that 4-PBA exhibits stage-specific effects on cardiac differentiation, whereas TM can activate JNK cascade to promote cardiac induction.

### 4-PBA and TSA exert stage-specific effects on cardiac differentiation

**Fig 2:**
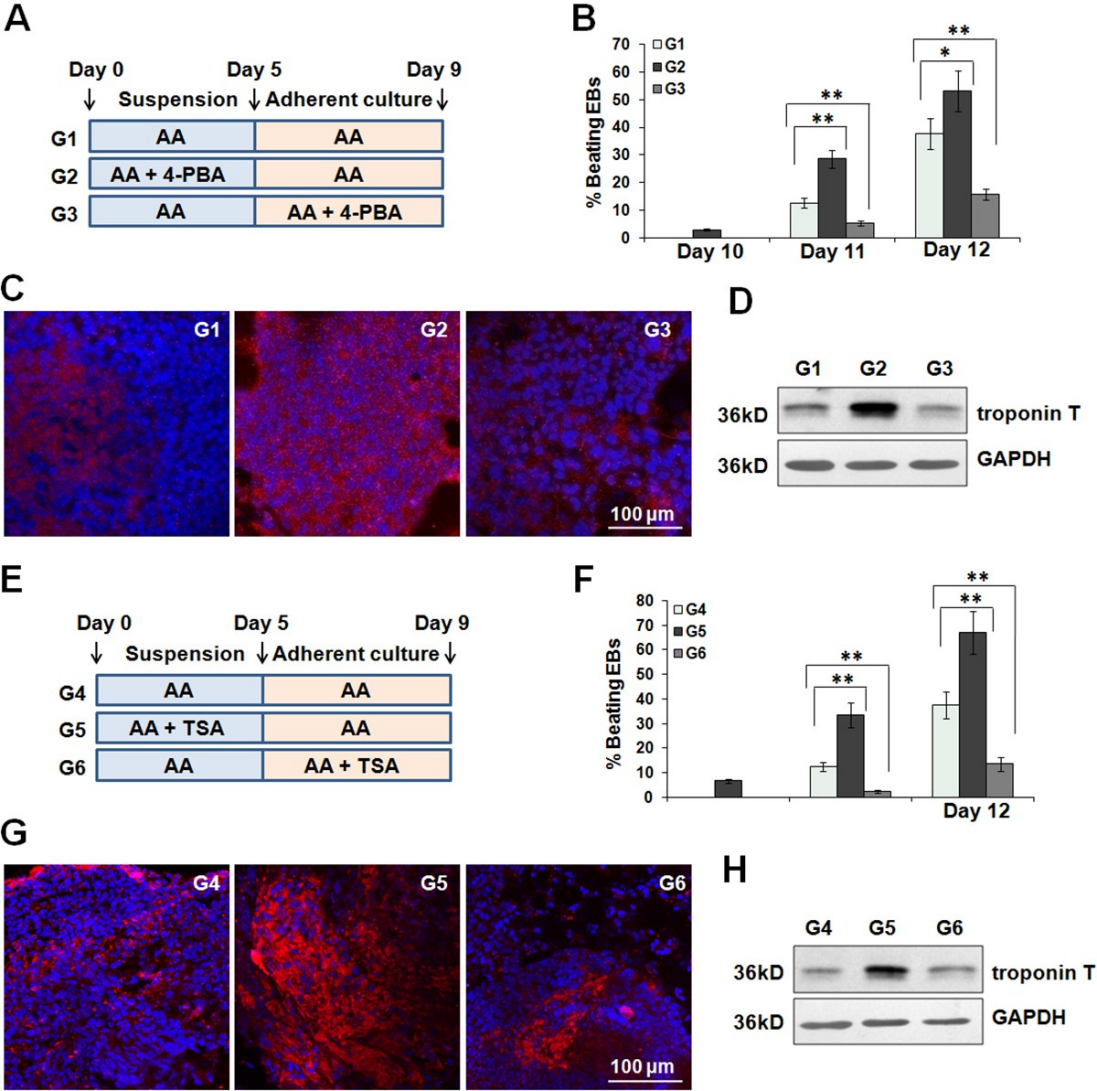
The effects of 4-PBA and TSA on the early- and late-stage cardiac differentiation. (A) Schematic diagram of differentiation protocol with 4-PBA treatment. (B) After 4-PAB treatment at indicated periods, the percentage of contracting EBs was calculated from day 10. *P<0.05, **P<0.01. (C) Immunofluorescence staining of troponin T (red) in control and 4-PBA groups at day 12. (D) Western blot showed the protein levels of troponin T among all groups at day 12. (E) Schematic diagram of differentiation protocol with TSA (10 nM) treatment. (F) The percentage of contracting EBs was calculated from day 10. *P<0.05, **P<0.01. (G) Immunofluorescence staining of troponin T (red) in control and TSA groups. (H) Western blot showed the protein levels of troponin T among all groups at day 12.

To examine the effect of 4-PBA on cardiac differentiation, 4-PBA was cultivated with AA-induced ES cells at the early or late stages (Fig 2A). The effect of 4-PBA on cardiomyogenesis was evaluated by assessment of the percentage of spontaneously beating embryoids. After 4-PBA treatment during the early stage, spontaneously beating EBs were found as early as day 9 and more percentage of EBs showed rhythmic contractions within the following days (Fig 2B). Obviously, 4-PBA promoted the early-stage cardiac differentiation, thereby further accelerated the generation of functional cardiomyocytes. Immunofluorescence assay also confirmed that 4-PBA treatment at the early stage could ultimately give rise to an enhancement in the yield of troponin T-positive cardiomyocytes at day 12 (Fig 2C). In parallel, similar results could be obtained by evaluating the expression of cardiac contractile troponin T protein (Fig 2D). In contrast, 4-PBA treatment during the late stage significantly inhibited cardiac differentiation, as demonstrated by decreased efficiency of beating EBs, reduced number of troponin T-positive cardiomyocytes.

Next, we speculated that 4-PBA might regulate cardiac differentiation in an ER stress-irrelevant manner. Besides ameliorating ER stress, 4-PBA also functions as a HDAC inhibitor. Subsequently, we estimated the stage-specific effects of a canonical pan-HDAC inhibitor TSA on cardiomyogenesis (Fig 2E). Intriguingly, TSA exhibited similar effects on cardiac differentiation. TSA treatment during the early stage promoted cardiac differentiation (Fig 2F–2H). While TSA treatment during the late stage inhibited the generation of spontaneously beating EBs and troponin T-positive cardiomyocytes. It is reasonable that 4-PBA and TSA might exert their effects to temporally promote or inhibit cardiomyogenesis via acetylation.

### The dynamic expression of HDACs and HATs during cardiomyogenesis

**Fig 3:**
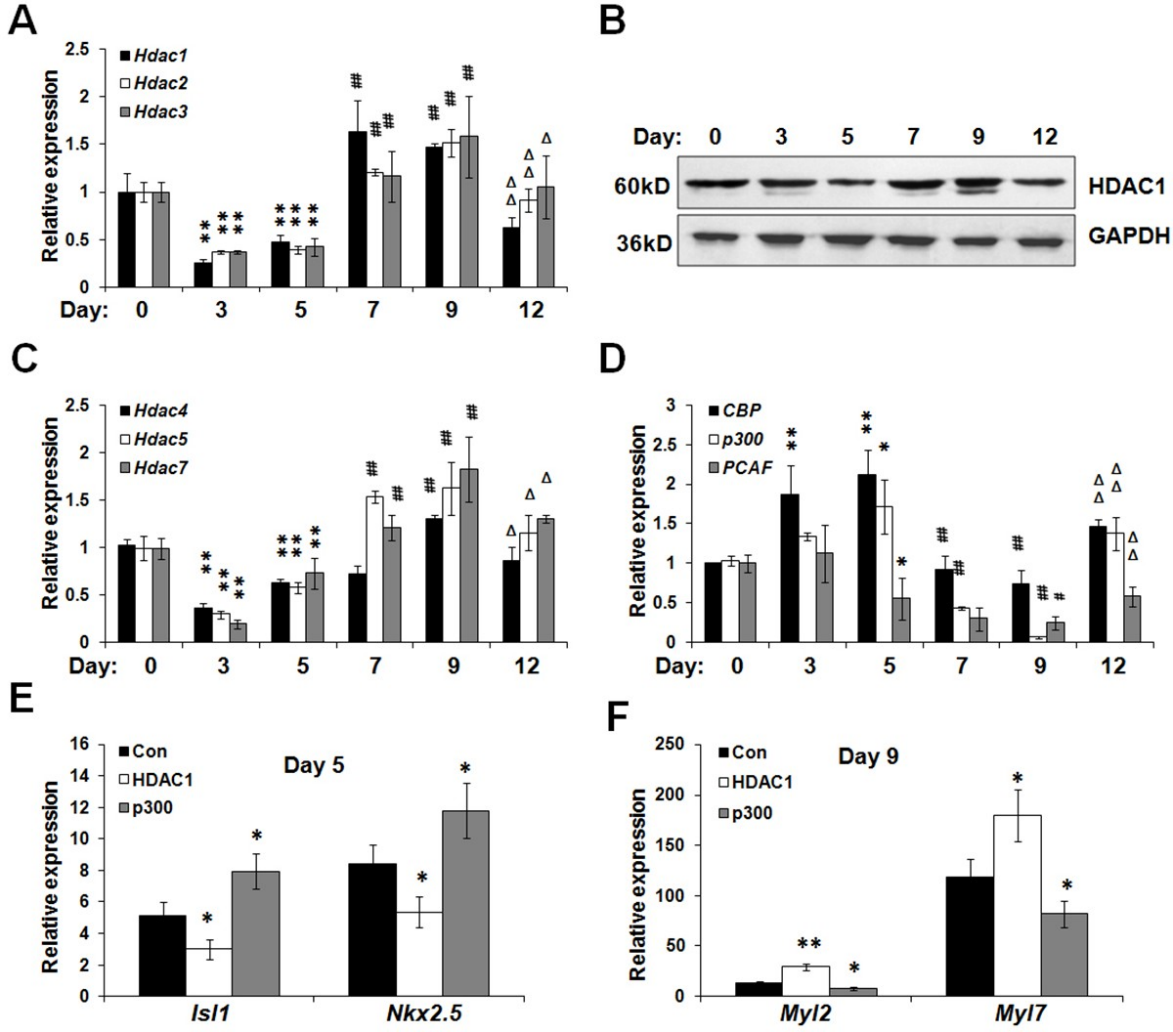
The stage-specific expression of HDAC1 during cardiomyogenesis. (A) Real-time PCR was conducted to evaluate the expression of Class I HDACs (*Hdac1*, *2*, and *3*) during cardiac induction. *P<0.05, **P<0.01 compared with data of Day 0. ^#^*P*<0.05, ^##^*P*<0.01 compared with data of Day 5. ^Δ^*P*<0.05, ^ΔΔ^*P*<0.01 compared with data of Day 9. (B) Western blot showed the protein alteration of HDAC1 during cardiac induction. (C) The expression fluctuation of Class IIa HDACs (*Hdac 4*, *5*, and *7*) during cardiomyogenesis was estimated by real-time PCR. *P<0.05, **P<0.01 compared with data of Day 0. ^#^*P*<0.05, ^##^*P*<0.01 compared with data of Day 5. ^Δ^*P*<0.05, ^ΔΔ^*P*<0.01 compared with data of Day 9. (D) The expression fluctuation of HATs such as *CBP*, *p300* and *PCAF*. *P<0.05, **P<0.01 compared with data of Day 0. ^#^*P*<0.05, ^##^*P*<0.01 compared with data of Day 5. ^Δ^*P*<0.05, ^ΔΔ^*P*<0.01 compared with data of Day 9. (E) ES cells at day 0 were transiently transfected with plasmids expressing HDAC1, p300 or GFP. After transfection, ES cells were allowed to form EBs and undergo cardiac differentiation in the presence of AA. Real-time PCR was conducted at day 5 to evaluate the expression of *Isl1* and *Nkx2*.*5*. (F) Differentiating ES cells at day 6 were transiently transfected with plasmids expressing HDAC1, p300 or GFP. After transfection, ES cells were allowed to further differentiation in adhesion. Real-time PCR was conducted at day 9 to evaluate the expression of *Myl2* and *Myl7*. *P<0.05, **P<0.01 compared with the control.

Subsequently, we investigated the dynamic expression of HDACs and HATs during cardiomyogenesis. As shown in Fig 3A, the expression of Class I HDACs (*Hdac1*, *2*, and *3*) displayed a sharp downregulation during the early-stage cardiac differentiation, while became upregulated during the late-stage cardiac differentiation. At day 12, the expression of Class I HDACs underwent a decrease. Western blot assay also validated that there was a downregulation of HDAC1 at day 3 and 5, while a relative upregulation at day 7 and 9, accompanied with a downregulation at day 12 (Fig 3B). Similarly, the expression of Class IIa HDACs (*Hdac4*, *5*, and *7*) firstly underwent a great drop at day 3, and increased at day 7 and 9 (Fig 3C), then mildly downregulated at day 12. CBP, p300 and PCAF function as HATs. *CBP* underwent a slight increase and *p300* maintained it expression level at day 3 and 5, both of their expression significantly decreased at day 7 and 9 (Fig 3D). Specially, p300 expression was dramatically downregulated at day 9 and upregulated at day 12. Collectively, these results suggested that HDACs and HATs exhibit distinct expression profiling during cardiomyogenesis.

Notably, HDAC1 exhibited an expression alteration totally different from p300 during cardiomyogenesis. Herein, we speculated that HDAC1 might be deleterious to mesodermal lineage commitment and the generation of cardiac progenitors, whereas it might play an indispensable role in the maturation of functional cardiomyocytes. Next, ES cells were transfected with plasmids encoding HDAC1, p300 or GFP and then were exposed to AA and allowed to form EBs. After cardiac induction for the early 5 days, EBs were subjected for RNA extraction and real-time PCR analysis. As shown in Fig 3E, forced expression of HDAC1 effectively decreased the expression of *Isl1* and *Nkx2*.*5*. By comparison, p300 overexpression enhanced the levels of *Isl1* and *Nkx2*.*5*. On the other hand, cardiac progenitors were transfected with HDAC1 or p300 at day 6. Obviously, HDAC1 overexpression directly increased the expression of *Myl2* and *Myl7* at day 9, while p300 did the opposite (Fig 3F). Overall, HDAC1 and p300 exert different roles at the distinct stages of cardiac induction.

### The dynamic expression and epigenetic modification of Oct4 during cardiomyogenesis

**Fig 4:**
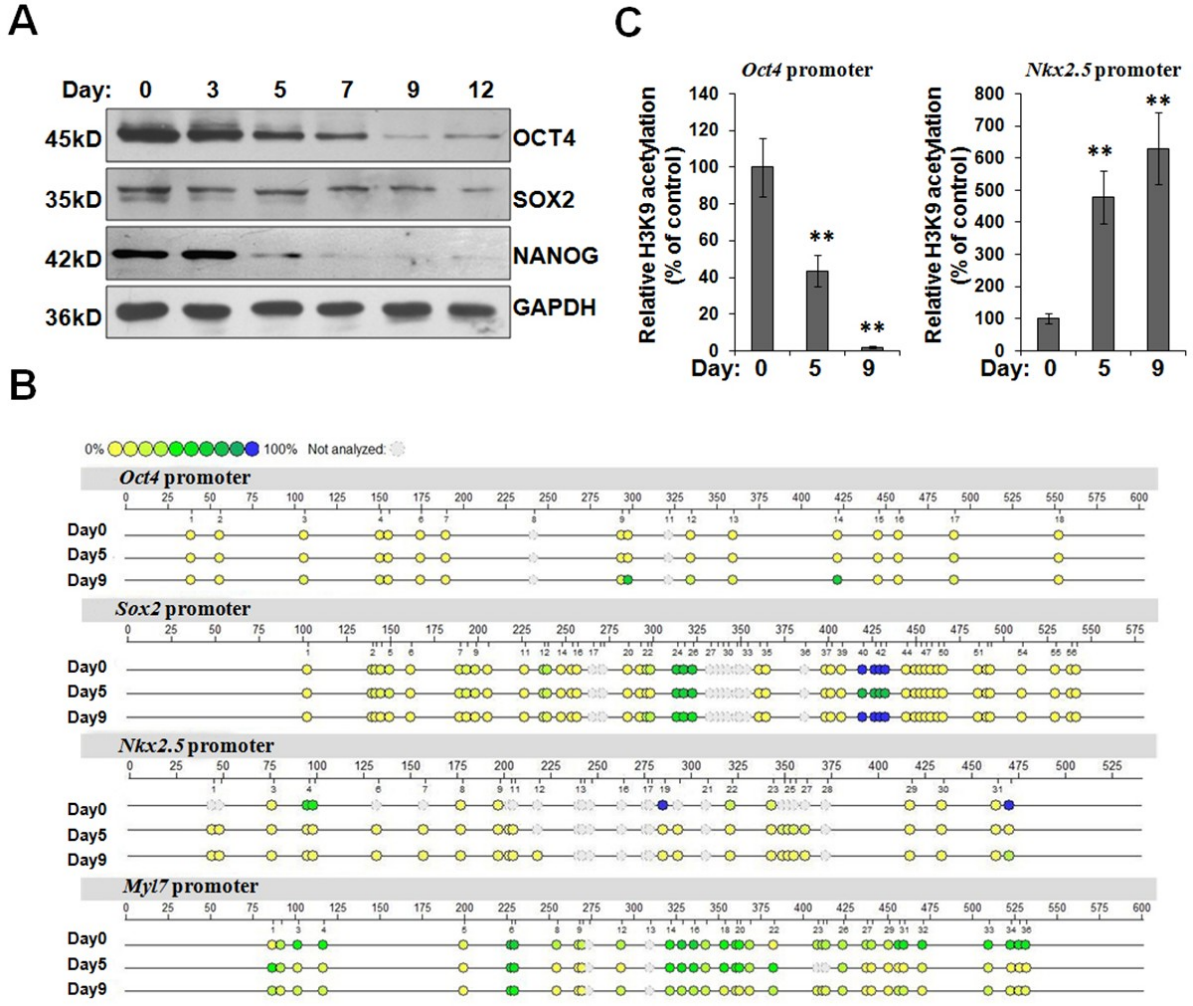
The stage-specific expression and epigenetic modification of *Oct4* during cardiomyogenesis. (A) Representative western blot analysis shows the expression kinetics of pluripotent genes in protein level during the process of cardiac induction. (B) Bisulfite genomic sequencing of the promoter regions of *Oct4*, *Sox2*, *Nkx2*.5, and *Myl7* in undifferentiated and differentiating ES cells. Different colors indicate the level of methylated CpGs. (C) ChIP assay was carried out to detect the levels of H3K9 acetylation on *Oct4* and *Nkx2*.*5* promoters. The immunoprecipitated DNA fragments were amplified by real-time PCR. 1% of the total input chromatin was used as internal control. Fold enrichment is the relative abundance of DNA fragments at the indicated promoters over internal control as quantified by real-time PCR. *P<0.05, **P<0.01 compared with the control.

It is feasible that HDAC1 upregulation during the late-stage cardiac differentiation might participate in the silence of pluripotency-associated genes, thereby facilitating cardiac differentiation. As shown in Fig 4A, OCT4 and NANOG, two transcription factors determining the pluripotent stem cell phenotype, declined from the early stage of differentiation and rapidly vanished during the late stage. Another pluripotent factor SOX2 exhibited a different expression profiling from OCT4 and NANOG.

Subsequently, we investigated the epigenetic modification of *Oct4* promoter during cardiomyogenesis. Bisulfite sequencing revealed that the cytosine guanine dinucleotides (CpG) on *Oct4* promoter were demethylated in ES cells, but became methylated in differentiating cells at day 9 (Fig 4B). Comparatively, the methylation on the CpG dinucleotides of *Nkx2*.5 promoter was effectively abolished during the early-stage cardiac differentiation. The promoter of *Myl7* showed densely methylated CpG dinucleotides in ES cells. Interestingly, several CpG sites on *Myl7* promoter were demethylated in differentiating cells at day 5, while others became unmethylated at day 9. Intriguingly, several CpG sites of *Sox2* promoter underwent a successive process of methylation and demethylation during cardiamyogenesis (Fig 4B). Indeed, our results strongly indicated that the conversion of *Oct4* gene from an accessible demethylated state to a condensed methylation one happened during the late-stage cardiac differentiation. Furthermore, we found that *Oct4* promoter in ES cells and differentiating cells at day 5 harbored high levels of H3K9 acetylation. When cells were transformed into immature or mature cardiomyocytes at day 9, the degree of H3K9 acetylation on *Oct4* promoter was significantly decreased (Fig 4C). By comparison, the enrichment of H3K9 acetylation on *Nkx2*.*5* promoter occurred during the early-stage cardiac differentiation. Taken together, the CpG methylation and H3K9 deacetylation of *Oct4* promoter is an important event of the late-stage cardiac differentiation.

### 4-PBA and TSA promote the early-stage cardiac differentiation via acetylation

**Fig 5:**
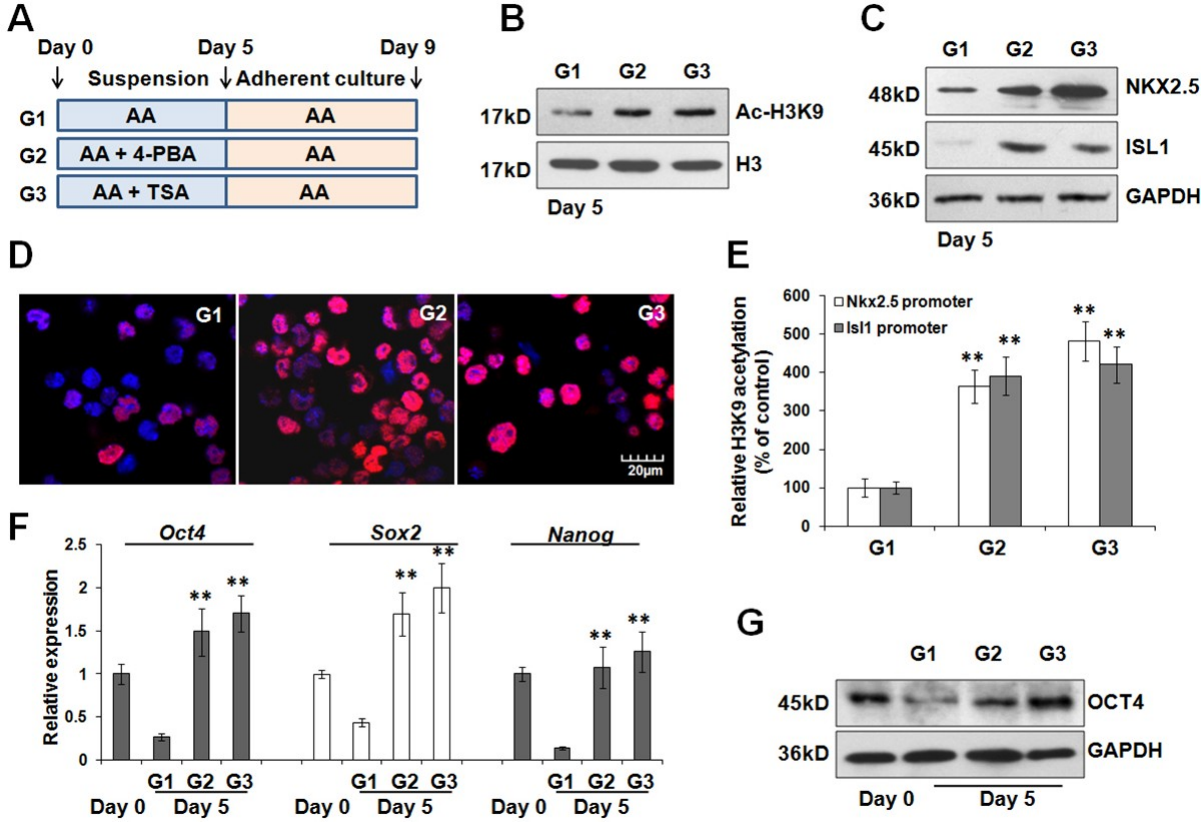
4-PBA and TSA promote the expression of cardiac transcription factors and OCT4 expression during the early-stage cardiac differentiation. (A) Schematic diagram of differentiation protocol with 4-PBA or TSA treatment during the early-stage cardiac differentiation. (B) Immunoblotting analysis for H3K9 acetylation following 4-PBA or TSA incubation. GAPDH was used as a loading control. (C) Western blot were performed at day 5 to detect the expression of ISL1 and NKX2.5 among all groups. (D) Immunofluorescence for NKX2.5 expression levels was performed with differentiating ES cells at day 5. Nuclei were counterstained with Hoechst33342. (E) ChIP assay was utilized to detect the levels of H3K9 acetylation on *Isl1* and *Nkx2*.*5* promoters after 4-PBA or TSA treatment during the early stage. **P<0.01 compared with the control. (F) Real-time PCR was conducted at day 5 to evaluate the expression of *Oct4*, *Nanog*, and *Sox2* after 4-PBA or TSA treatment. **P<0.01 compared with G1 group. (G) The amount of OCT4 protein after 4-PBA or TSA treatment was assessed at day 5 by immunoblotting analysis.

Based on these findings above, we speculated that 4-PBA and TSA affect different gene program during the early- or late-stage cardiac differentiation. As showed in Fig 5A, ES cells were treated with 4-PBA or TSA from the beginning to day 5. Evidently, the treatment of 4-PBA or TSA significantly increased the acetylation of histone H3 at lysine 9 (H3K9) (Fig 5B). As a result, the expression levels of ISL1 and NKX2.5 were significantly enhanced (Fig 5C). Immunofluorescence assay also confirmed a significant expansion of NKX2.5-positive cardiac progenitor cells at day 5 (Fig 5D). Consistently, 4-PBA and TSA administration resulted in increased H3K9 acetylation on *Isl1* and *Nkx2*.*5* promoters (Fig 5E). In parallel, we also observed that the downregulation of pluripotency genes *Oct4*, *Nanog*, and *Sox2* was markedly attenuated by 4-PBA or TSA treatment (Fig 5F). Immunoblotting assay further validated that 4-PBA or TSA treatment significantly hindered the depletion of OCT4 protein during the early stage (Fig 5G). Obviously, our results suggested that 4-PBA and TSA administration promotes the early-stage cardiac differentiation and induces the expression of cardiac transcription factors via acetylation. Simultaneously, acetylation also maintains an active chromatin status of *Oct4*. Seemingly, OCT4 expression does not interfere in the induction of cardiac transcription factors as markers of the early-stage cardiac differentiation.

### 4-PBA and TSA attenuate the late-stage cardiac differentiation via acetylation

**Fig 6:**
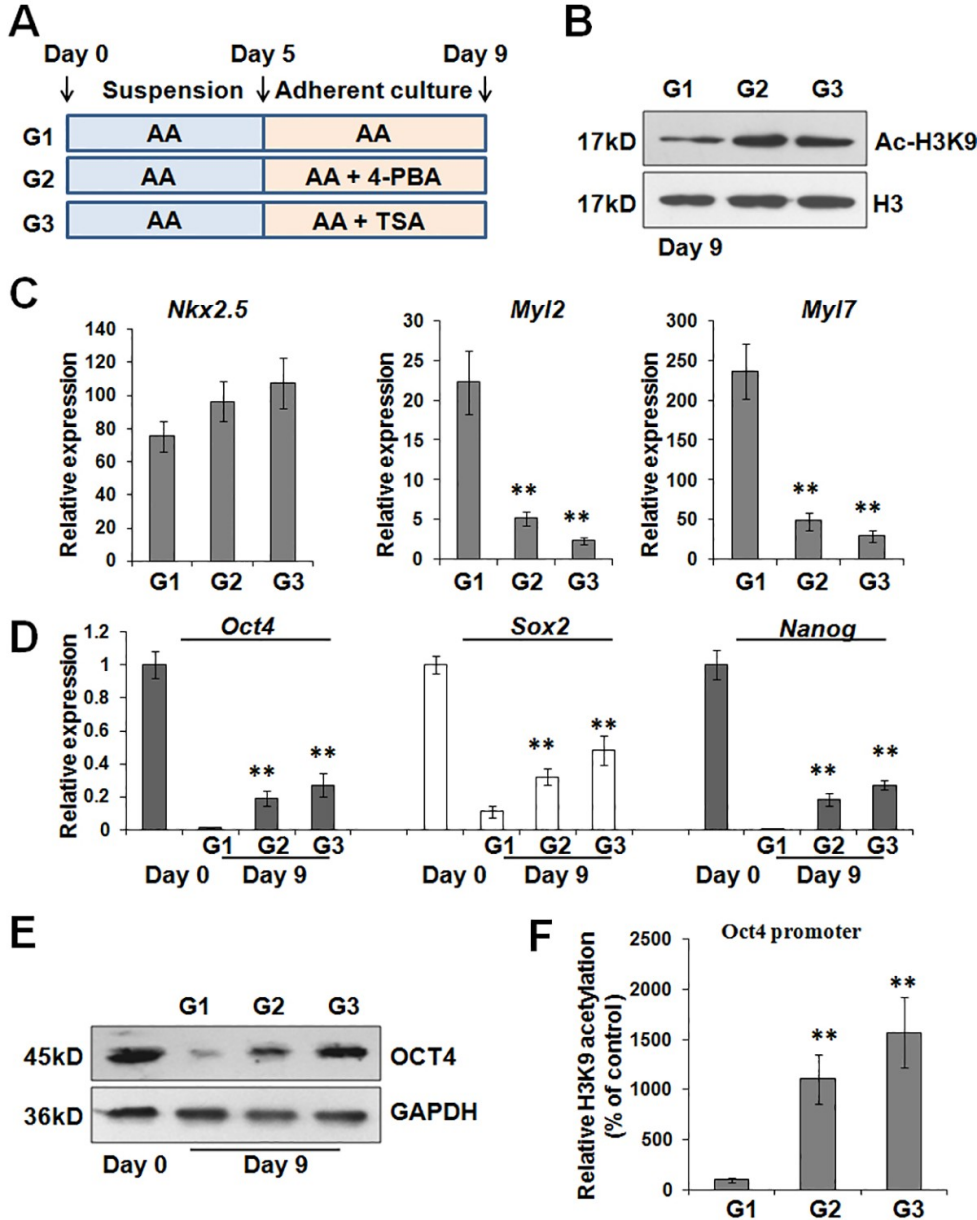
4-PBA and TSA inhibit the expression of cardiac functional genes and maintain OCT4 expression during the late-stage cardiac differentiation. (A) Schematic diagram of differentiation protocol with 4-PBA or TSA treatment during the late-stage cardiac differentiation. (B) Immunoblotting analysis for H3K9 acetylation following 4-PBA or TSA incubation. (C) After 4-PBA or TSA treatment during the late stage, the expression of *Nkx2*.5, *Myl2*, and *Myl7* at day 9 was estimated by real-time PCR. **P<0.01 compared with the control. (D) Real-time PCR was conducted at day 9 to evaluate the expression of *Oct4*, *Nanog*, and *Sox2* after 4-PBA or TSA treatment. **P<0.01 compared with G1 group. (E) The amount of OCT4 protein after 4-PBA or TSA treatment was assessed at day 9 by immunoblotting analysis. (F) ChIP assay was utilized to detect the levels of H3K9 acetylation on *Oct4* promoter after 4-PBA or TSA treatment during the late stage of cardiac induction. **P<0.01 compared with the control.

Similarly, we treated cells with 4-PBA and TSA during the late-stage cardiac differentiation (Fig 6A). Undoubtedly, the treatment of 4-PBA or TSA enhanced the acetylation of histone H3 (Fig 6B). As shown in Fig 6C, 4-PBA or TSA treatment greatly decreased the expression of *Myl2* and *Myl7* at day 9. At the same time, the gene silence of pluripotency genes *Oct4*, *Nanog*, and *Sox2* was markedly impeded by 4-PBA or TSA treatment (Fig 6D). Immunoblotting assay further confirmed that 4-PBA and TSA retarded the decrease of OCT4 protein during the late stage (Fig 6E). As expected, ChIP assay showed that 4-PBA or TSA treatment led to higher levels of H3K9 acetylation on *Oct4* promoter (Fig 6F). Thus, these results implicated a possibility that acetylation might deteriorate the late-stage cardiac differentiation via maintaining a high level of redundant OCT4 protein.

### 4-PBA and TSA favor the maturation of cardiomyocytes

**Fig 7:**
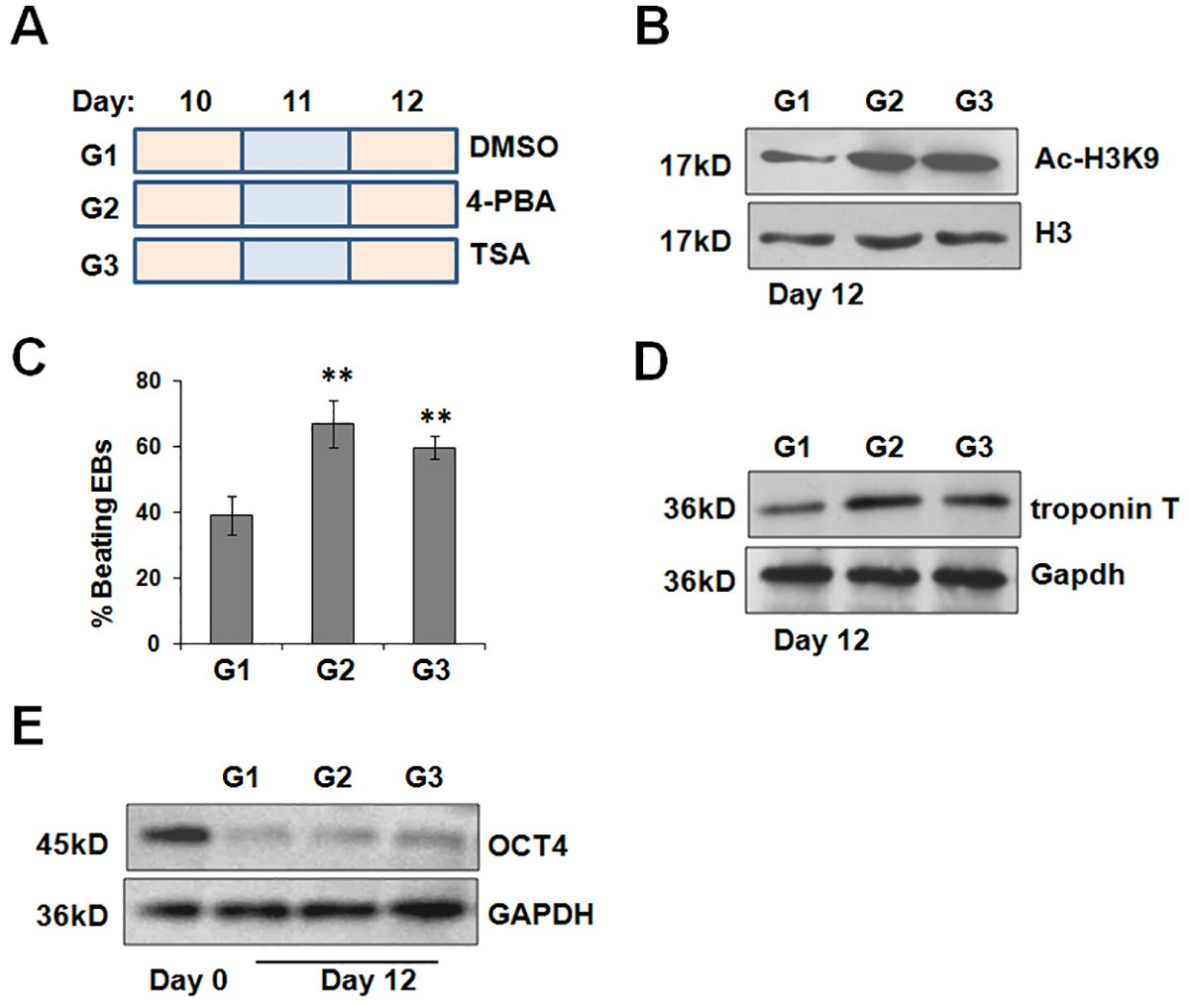
4-PBA and TSA treatment from day 10 to day 12 increase the generation of contractile EBs. (A) Schematic diagram of differentiation protocol with 4-PBA or TSA treatment from day 10 to day 12. Culture medium should be changed daily. (B) Immunoblotting analysis for H3K9 acetylation following 4-PBA or TSA incubation. (C) The percentage of beating EBs in 4-PBA or TSA treatment groups was assessed at day 12. **P<0.01 compared with the control. (D) Western blot showed the protein levels of troponin T among all groups at day 12. (E) The amount of OCT4 protein after 4-PBA or TSA treatment was assessed at day 12 by immunoblotting analysis.

Subsequently, we estimated the effects of 4-PBA and TSA after *Oct4* expression is effectively silenced in differentiating cardiomyocytes. Thus, cells were exposed to 4-PBA and TSA from day 10 to day 12 (Fig 7A). 4-PBA or TSA treatment led to an increase in H3K9 acetylation (Fig 7B). Interestingly, 4-PBA or TSA treatment enhanced the percentage of contractile EBs and increased the expression of troponin T (Fig 7C and 7D). Relatively, the OCT4 expression did not effectively rescued by acetylation (Fig 7E). It seemed that acetylation promotes the maturation of differentiating cardiomyocytes when these cells exit from the pluripotent state.

## Discussion

For cardiomyogenesis, stem cells are induced to undergo a consecutive array of differentiation, including mesoderm formation, specification of cardiac mesodermal cells towards cardiac progenitors, and the elaboration of functional beating cardiomyocytes. DNA methylation and histone modifications play a crucial role in cardiac lineage differentiation and specification, temporally and spatially modulating the complicated gene programs. 4-PBA, is a molecular inhibitor of ER stress and HDACs. Herein, our results showed that 4-PBA regulates cardiac differentiation via modulating histone acetylation.

Histone acetylation mediated by HATs is a reversible process characterized by transferring the acetyl moiety from acetyl co-enzyme A to lysine residues, thereby relaxing chromatin structure and facilitating transcription factors access to DNA. Conversely, HDACs remove acetyl groups from the N-acetylated lysine residues on histones and strengthen histone-DNA binding to repress gene expression. p300 is the most studied HAT in cardiac development. p300 is highly expressed in embryonic myocardium while its level declines after birth. p300 deletion in mice led to lethality at embryonic day 9–11.5 accompanied with heart malformations and reduced expression of cardiac-specific genes [18]. As an epigenetic machinery in the differentiation of ES cells, Gata4 recruits p300 to establish H3K27Ac domains in mesodermal cells [19].

Mammalian genome encodes 18 HDACs, which can be divided into four classes based on the homology with yeast histone deacetylases. Class Ⅰ HDACs comprise of HDAC1, -2, -3 and -8, which are ubiquitously expressed in many tissues and cell lines. The deacetylation of histones to repress gene transcription mainly attributes to the function of class Ⅰ HDACs. The importance of HDACs also has been demonstrated for cardiac differentiation *in vitro* and heart development *in vivo*. Mouse embryos deficient in *Hdac1* die at embryonic day 9.5, whereas mice lacking *Hdac2* survive until the perinatal period and succumb to a spectrum of severe cardiac defects [20]. Another study showed that loss of *Hdac1*, but not *Hdac2*, reduced the level of HDAC activity in corepressor complexes and led to enhanced differentiation of both cardiomyocytes and neuronal cells [21]. Meanwhile, the expression levels of HDAC1 and 3 decreased gradually along with differentiation. Ectopic expression of HDAC1 or 3 significantly hindered differentiation of ES cells into the mesodermal lineage [22]. The activities of HDACs also regulate entry of mesoderm cells into the cardiac muscle lineage [23]. *Hdac1* depletion stimulated cardiomyocyte formation, increased the levels of acetylated lysines and upregulated the expressions of GATA4 and NKX2.5 in ES cells and embryonal carcinoma cells [24, 25]. These findings established a unique requirement for HDAC1 in cell fate determination and cardiac differentiation.

Several studies further demonstrated the potential merit of HDAC inhibition in manipulating stem cell differentiation into cardiac fates. TSA stimulation facilitated myocardial differentiation of monkey ES cells [26]. Another study showed that TSA over a narrow window of concentrations directed the differentiation of human iPS cells into cardiomyocytes [27]. TSA also augmented the acetylated form of GATA4 and its DNA binding during the ES cell differentiation, facilitating the expression of endogenous cardiac β-myosin heavy chain [28].

Paradoxically, there is also opposite evidence that the loss of HDAC1 counteracted cardiac differentiation of both ESCs and iPSCs [29]. Herein, our results help to resolve some of the discrepancies between these studies, indicating that HDACs exert stage-specific effects on cardiomyogenesis. Firstly, we found stage-specific fluctuation in the expression of HDAC1. HDAC1 displayed a sharp downregulation during the early-stage cardiac differentiation, while became upregulated during the late-stage cardiac differentiation. Secondly, forced expression of HDAC1 during the early stage of cardiac induction effectively decreased the expression of *Isl1* and *Nkx2*.*5*. Whereas HDAC1 overexpression during the late stage directly increased the expression of *Myl2* and *Myl7*. Thus, our results strongly support that HDAC1 exert different roles at the distinct stages. As a consequence, 4-PBA and TSA impose stage-specific effects to regulate cardiac differentiation via inhibition of HDACs.

Further investigation revealed that the stage-specific effects of HDAC inhibitors might attribute to the temporal fluctuation of transactivational and epigenetic status on pluripotency-associated genes. In this study, we found that the epigenetic choreography of cardiac differentiation was composed by sequential phases and hierarchical mode of DNA methylation and histone acetylation on the promoters of *Oct4*, *Nkx2*.*5*, and *Myl7* genes. OCT4 declined from the early stage of differentiation and rapidly vanished during the late stage. Notably, the CpG methylation and H3K9 deacetylation of *Oct4* promoter mainly happened during the late stage of cardiac differentiation. Meanwhile, the majority of CpG sites on *Myl7* promoter were demethylated during the late stage. Nevertheless, the transactivation of NKX2.5 was an event of the early differentiation stage. Similar to our results, previous studies suggested that the promoter regions of cardiac-specific transcription factors are mainly demethylated in both ES cells and cardiomyocytes. However, the promoters of the genes encoding cardiac structural proteins are demethylated only in cardiomyocytes derived from ES cells [30].

In this study, we found that 4-PBA and TSA treatment during the early stage enhanced the expression of ISL1 and NKX2.5, accompanied by interrupted downregulation in OCT4 expression. Seemingly, high levels of OCT4 did not exert detrimental effects on the early-stage differentiation. Several evidences indicated that OCT4 was required for BMP4-induced mesendoderm induction, and NANOG is essential for mesoderm induction by both BMP4/activin A and GSK3β inhibitors [31, 32]. OCT4 could regulate specification of embryonic stem cells toward a cardiac lineage in a dose-dependent manner [33]. Another study showed that HDAC inhibition can promote ES cell self-renewal across species [34]. Butyrate, a HDAC inhibitor, stimulated the expression of a group of pluripotency-associated genes during reprogramming [35]. In contrast, catalytic inhibition of CBP/p300 prevented iPSC formation [36]. Importantly, high levels of pluripotency-associated genes seemed to be adverse to the late-stage differentiation, perhaps inhibiting the initiation of cardiac structural and contractile genes such as *Myl7*, a cardiomyocyte maturation/atrium-specific marker. However, the precise mechanism remains to be elucidated.

Interestingly, 4-PBA or TSA treatment after the occurrence of spontaneous rhythmic contraction enhanced the percentage of contractile EBs and increased the expression of troponin T. Simultaneously, OCT4 expression did not validly rescued by HDAC inhibition. Thus, we assumed that acetylation promotes the maturation of differentiating cardiomyocytes when these cells exit from the pluripotent state. This finding further validates our hypothesis that the stage-specific effects of HDAC inhibitors might be due to the expression status of pluripotency-associated genes. On the other hand, TSA and other HDAC inhibitors could attenuate pathological cardiac remodeling and hypertrophic gene expression in adult cardiomyocytes [37]. Thus, cardiomyocytes may undergo another alteration in responsiveness to HDAC inhibitors after birth.

## Conclusions

Our results indicated 4-PBA and TSA as HDAC inhibitors exert stage-specific effects on cardiac differentiation. And the context-dependent effects of HDAC inhibitors depend on cell differentiation states marked by the temporal expression and silence of pluripotency-associated genes.

## Supporting information

S1 File (DOCX)

S1 Table Primers used for real-time PCR. (DOC)

S2 Table Primers used for Bisulfite sequencing. (DOC)

S3 Table Primers used for ChIP assay. (DOC)
