# Supplementary material for: 4-phenylbutyrate exerts stage-specific effects on cardiac differentiation via HDAC inhibition
Source: PLoS One. 2021 Apr 21;16(4):e0250267. doi: 10.1371/journal.pone.0250267 (PMC8059837; doi:10.1371/journal.pone.0250267)
Supplement: S1 File — (DOCX) [file pone.0250267.s001.docx]

**Fig1G**

**p-JNK**

**Con 4-PBA TM**

**JNK**

**Con 4-PBA TM**

**Fig2D**

**Troponin T**

**G1 G2 G3**

**GAPDH**

**G1 G2 G3**

**Troponin T**

**G4 G5 G6**

**GAPDH**

**G4 G5 G6**

**Fig3B**

**HDAC1**

**Day: 0 3 5 7 9 12**

**GAPDH**

**Day: 0 3 5 7 9 12**

**Fig4A**

**OCT4**

**Day: 0 3 5 7 9 12**

**SOX2**

**Day: 0 3 5 7 9 12**

**NANOG**

**Day: 0 3 5 7 9 12**

**GAPDH**

**Day: 0 3 5 7 9 12**

**Fig5B**

**Ac-H3K9**

**G1 G2 G3**

**H3**

**G1 G2 G3**

**Fig5C**

**NKX2.5**

**G1 G2 G3**

**ISL1**

**G1 G2 G3**

**GAPDH**

**G1 G2 G3**

**Fig5G**

**OCT4**

**Day 0 G1 G2 G3**

**GAPDH**

**Day 0 G1 G2 G3**

**Fig6B**

**Ac-H3K9**

**G1 G2 G3**

**H3**

**G1 G2 G3**

**Fig6E**

**OCT4**

**Day 0 G1 G2 G3**

**GAPDH**

**Day 0 G1 G2 G3**

**Fig7B**

**Ac-H3K9**

**G1 G2 G3**

**H3**

**G1 G2 G3**

**Fig7D**

**troponin T**

**G1 G2 G3**

**GAPDH**

**G1 G2 G3**

**Fig7E**

**OCT4**

**Day 0 G1 G2 G3**

**GAPDH**

**Day 0 G1 G2 G3**
