# Supplementary material for: 4-phenylbutyrate exerts stage-specific effects on cardiac differentiation via HDAC inhibition
Source: PLoS One. 2021 Apr 21;16(4):e0250267. doi: 10.1371/journal.pone.0250267 (PMC8059837; doi:10.1371/journal.pone.0250267)
Supplement: S1 Table — (DOC) [file pone.0250267.s002.doc]

**S1 Table Primers used for real-time PCR**

| **Gene** | **Primer Sequence(5’ to 3’)** | **AT(°C)** | **Product(bp)** |
| --- | --- | --- | --- |
| *Isl1* | F-CAGTCCCAGAGTCATCCGAGT  R-TGGGTTAGCAGTTTTGTCGTT | 58 | 115 |
| *Nkx2.5* | F-CAAGTGCTCTCCTGCTTTCC  R- GGCTTTGTCCAGCTCCACT | 58 | 136 |
| *Myl2* | F-AGGACGAGTGAACGTGAAAAAT  R-ACACGGTGAAGTTAATTGGACC | 58 | 77 |
| *Myl7* | F-GGCACAACGTGGCTCTTCTAA  R- GATTTGCAGATGATCCCATCCC | 58 | 114 |
| *Oct4* | F-AGTTGGCGTGGAGACTTTGC  R-CAGGGCTTTCATGTCCTGG | 58 | 160 |
| *Sox2* | F-GCGGAGTGGAAACTTTTGTCC  R-CGGGAAGCGTGTACTTATCCTT | 58 | 157 |
| *Nanog* | F-CCTGATTCTTCTACCAGTCCCA  R-GGCCTGAGAGAACACAGTCC | 58 | 123 |
| *Caspase-3* | F-ATGGAGAACAACAAAACCTCAGT  R-TTGCTCCCATGTATGGTCTTTAC | 58 | 74 |
| *Caspase-8* | F-TGCTTGGACTACATCCCACAC  R-TGCAGTCTAGGAAGTTGACCA | 58 | 169 |
| *Caspase-9* | F-TCCTGGTACATCGAGACCTTG  R-AAGTCCCTTTCGCAGAAACAG | 58 | 101 |
| *Chop* | F-CTGGAAGCCTGGTATGAGGAT  R-CAGGGTCAAGAGTAGTGAAGGT | 58 | 121 |
| *Bip* | F-ACTTGGGGACCACCTATTCCT  R-ATCGCCAATCAGACGCTCC | 58 | 134 |
| *Xbp1* | F-AGCAGCAAGTGGTGGATTTG  R-GAGTTTTCTCCCGTAAAAGCTGA | 58 | 75 |
| *Atf4* | F-CCTGAACAGCGAAGTGTTGG  R-TGGAGAACCCATGAGGTTTCAA | 58 | 134 |
| *Hdac1* | F-AGTCTGTTACTACTACGACGGG  R-TGAGCAGCAAATTGTGAGTCAT | 58 | 101 |
| *Hdac2* | F-GGAGGAGGCTACACAATCCG  R-TCTGGAGTGTTCTGGTTTGTCA | 58 | 173 |
| *Hdac3* | F-GCCAAGACCGTGGCGTATT  R-GTCCAGCTCCATAGTGGAAGT | 58 | 61 |
| *Hdac4* | F-CACTGCATTTCCAGCGATCC  R-AAGACGGGGTGGTTGTAGGA | 58 | 113 |
| *Hdac5* | F-AGCACCGAGGTAAAGCTGAG  R-GCTGTGGGAGGGAATGGTT | 58 | 91 |
| *Hdac7* | F-GGCAGGCTTACACCAGCAA  R-TGGGCAGGCTGTAGGGAATA | 58 | 254 |
| *CBP* | F-GGCTTCTCCGCGAATGACAA  R-GTTTGGACGCAGCATCTGGA | 58 | 136 |
| *p300* | F-AGCCAAGCGGCCTAAACTC  R-CGCCACCATTGGTTAGTCCC | 58 | 143 |
| *PCAF* | F-CATCTCAGCGGAGACTACGG  R-ACATTGCAGTAGCACAACCAC | 58 | 85 |
| *18S rRNA* | F- GTAACCCGTTGAACCCCATT  R-CCATCCAATCGGTAGTAGCG | 58 | 151 |
