# Supplementary material for: 4-phenylbutyrate exerts stage-specific effects on cardiac differentiation via HDAC inhibition
Source: PLoS One. 2021 Apr 21;16(4):e0250267. doi: 10.1371/journal.pone.0250267 (PMC8059837; doi:10.1371/journal.pone.0250267)
Supplement: S2 Table — (DOC) [file pone.0250267.s003.doc]

**S2 Table Primers used for Bisulfite Sequencing**

| **promoter** | **Primer Sequence(5’ to 3’)** |
| --- | --- |
| *Oct4* | F-aggaagagagATTTAAGGTAGGGGTGAGAGGATTT  R-cagtaatacgactcactatagggagaaggctCCAACCTAAAATCCACAATATACCA |
| *Sox2* | F-aggaagagagTGTAAAAAGGGAAAAGTATTTTGTTG  R-cagtaatacgactcactatagggagaaggctCATCTTAAAATTCTCCTAAACCATCTT |
| *Nkx2.5* | F-aggaagagagGTTAGGGAAATAGATGGGGGTTTT  R-cagtaatacgactcactatagggagaaggctCTCCCAAATTCTCTTTAACAACAAA |
| *Myl7* | F-aggaagagagTTTTTGAAGTTGAATAGGAGGAAAA  R-cagtaatacgactcactatagggagaaggctTAAACAAAACAAAACATTCCCATTC |
