# Supplementary material for: 4-phenylbutyrate exerts stage-specific effects on cardiac differentiation via HDAC inhibition
Source: PLoS One. 2021 Apr 21;16(4):e0250267. doi: 10.1371/journal.pone.0250267 (PMC8059837; doi:10.1371/journal.pone.0250267)
Supplement: S3 Table — (DOC) [file pone.0250267.s004.doc]

**S3 Table Primers used for ChIP assay**

| **promoter** | **Primer Sequence(5’ to 3’)** | **AT(°C)** | **Product(bp)** |
| --- | --- | --- | --- |
| *Isl1* | F-GGTGAATGCCTGTATATGTTTGGA  R- AAATAGGGGATCTGAACCAATTCT | 60 | 138 |
| *Nkx2.5* | F-ttgcaaaggaaaCGtcccct  R-tgtgagctgaCGcctgaaat | 60 | 122 |
| *Oct4* | F-AGCCCCACTAAACAAAGCAC  R-GCAATCCCCTCAAAGACTGA | 60 | 161 |
